# Optimizing *Valerianella locusta* L. Growth and Metabolism by Combining Red and Blue LED Light: Insights into Plant Physiology, Biochemistry, and Nutraceutical Value

**DOI:** 10.3390/plants14121887

**Published:** 2025-06-19

**Authors:** Sonia Monterisi, Carmen Rebollo Vicioso, Monica Yorlady Alzate Zuluaga, Sofia Melchior, Biancamaria Senizza, Gokhan Zengin, Roberto Fattorini, Umberto Lanza, Talita de Oliveira Caretta, Lara Manzocco, Luigi Lucini, Stefano Cesco, Youry Pii

**Affiliations:** 1 Faculty of Agricultural, Environmental and Food Sciences, Free University of Bozen/Bolzano, Piazza Università 1, 39100 Bolzano, Italymonicayorlady.alzatezuluaga@unibz.it (M.Y.A.Z.); roberto.fattorini@student.unibz.it (R.F.); talita.deoliveiracaretta@unibz.it (T.d.O.C.); stefano.cesco@unibz.it (S.C.); 2 Department of Agricultural, Food, Environmental and Animal Sciences, University of Udine, Via Sondrio 2/A, 33100 Udine, Italy; sofia.melchior@uniud.it (S.M.); lanza.umberto@spes.uniud.it (U.L.); lara.manzocco@uniud.it (L.M.); 3 Department of Human Sciences and Promotion of the Quality of Life, San Raffaele University, 00166 Rome, Italy; 4 Department for Sustainable Food Process, Università Cattolica del Sacro Cuore, 29122 Piacenza, Italy; biancamaria.senizza@unicatt.it (B.S.); luigi.lucini@unicatt.it (L.L.); 5 Department of Biology, Science Faculty, Selcuk University Campus, Konya 42130, Turkey; gokhanzengin@selcuk.edu.tr

**Keywords:** lamb’s lettuce (*Valerianella locusta* L.), organic acids, phenolic compounds, antioxidant activity, metabolomics, shelf-life

## Abstract

Environmental and health concerns have increased the demand for ready-to-eat vegetables rich in bioactive compounds. This study explores the impact of red and blue (R:B) LED light on the metabolic responses of lamb’s lettuce (*Valerianella locusta* L.), focusing on sugars, organic acids, total phenolics, antioxidant activity, and enzyme inhibition. Post-harvest analyses were also conducted to assess shelf-life and microbiological characteristics of the product. The R:B LED treatment significantly enhanced plant growth, with a 133% and 68% increase in shoot fresh and dry weights, respectively, and a 21% increase in leaf area compared to controls (white LED light). Biochemical profiling revealed substantial increases in fructose (255%), sucrose (169%), citric acid (350%), and malic acid (868%) under R:B LED light. Additionally, phenolic content increased by 30%, alongside a notable modulation of 258 secondary metabolites, including flavonoid glycosides, alkaloids, and terpenoids. These biochemical changes contributed to a marked improvement in antioxidant capacity (12–45% across multiple assays) and a 300% increase in α-glucosidase inhibition, suggesting potential antidiabetic properties. Furthermore, post-harvest analysis revealed comparable shelf-life and microbiological safety between R:B and white LED-grown samples. The research highlights the potential of LED light to enhance plant biochemical responses and improve crop quality without affecting post-harvest quality, paving the way for sustainable agricultural innovations.

## 1. Introduction

The United Nations Food and Agriculture Organization’s (FAO) estimated that between 691 and 783 million people in the world faced hunger in 2022 and more than 3.1 billion people were unable to afford healthy diet in 2021 [1]. Additionally, about two billion individuals experienced micronutrient malnutrition (MNM) or “hidden hunger”, which is a form of hunger resulting from insufficient vital micronutrients daily dietary intake [2]. Today, new trends in agriculture are therefore aimed at growing nutrient-rich food crops to counter MNM rather than focusing solely on increasing yields.

Indeed, improving the bioavailability of essential minerals, vitamins, and antioxidants in crops is becoming increasingly important for human health and disease prevention. Within this framework, plant secondary metabolites—especially polyphenols, flavonoids, and other antioxidants—are key to developing more nutritious crops, helping address the low nutritional quality of Western diets. Many studies now assess antioxidant activity in food and plant samples as an initial screening step [3], since antioxidants protect against diseases linked to oxidative stress, including cancer, cardiovascular, and neurodegenerative disorders [4]. Among these substances, phenolic compounds (e.g., flavonoids, phenolic acids, and tannins) are widespread in plants and act as powerful antioxidants [5]. Indeed, these together with ascorbic acid, carotenoids, and vitamin E counteract oxidative damage by neutralizing free radicals and ROS [6,7]. To boost these health-promoting compounds in crops, various agronomic strategies have been explored, especially light-based approaches that influence phenolic compound synthesis [8,9]. A recent study demonstrated the significant impact of the light-emitting diode (LED) on the levels of sulphur compounds, polyphenols, and the antioxidant potential of the biomass extracts of watercress plants [10]. A LED is a semiconductive diode that can produce light through electroluminescence, which emits a narrow spectrum of light in a non-coherent manner. Distinct wavelengths of light are emitted, giving rise to monochromatic light with specific visible colours [11]. The colour of the emitted light is determined by the band gap energy of the semiconductor material used [12]. The capability of modulating monochromatic light makes the use of LEDs interesting for plant applications. In fact, the growth and development of plants are significantly influenced by light intensity and its spectral properties, with red and blue light being considered the most important ones [13]. Red (R) wavelengths (600–700 nm) and blue (B) wavelengths (400–500 nm) can elicit higher CO_2_ assimilation or O_2_ production rates in plants, with R light being particularly efficient [14]. B light influences plant growth, leaf expansion, photomorphogenesis, stomatal opening, photosynthesis, and pigment accumulation [15]. Moreover, the utilization of B LED light stimulates the biosynthesis of both primary and secondary metabolites in plants [16]. On the other hand, R light plays a crucial role in regulating chloroplast function, stem and petiole growth, as well as the reproductive system [15]. Furthermore, it was demonstrated that R light can also effectively reduce nitrate levels in plants [17]. Notwithstanding this, different studies revealed that either monochromatic R or B light is insufficient to meet the normal growth requirements of plants. Indeed, plants showed photosynthetic inefficiencies when one of these light qualities was absent [18]. Conversely, the combined use of R and B LEDs to irradiate plants showed synergetic effects inducing improvements in fresh mass and dry mass content of cucumber compared to R LEDs alone [18]. Similarly, the combination of R and B LED lights led to a significant increase of 161% in the dry weight of plants, as concluded in a recent meta-analysis [19]. Moreover, the synergistic effect of B and R light was demonstrated to enhance pigment contents, growth, and antioxidant activity of kale, basil, lettuce, spinach, and sweet pepper [20]. A study conducted on two varieties of *Ocimum basilicum* reported that a treatment involving a 2.3:1 R:B LED combination led to enhanced synthesis of phenols and carotenoids [21]. Moreover, different R:B LED combination treatments resulted in an elevation of the overall antioxidant pool in basil with an observed 70% increase in flavonoids across all LED treatments [22]. Besides their significant role in plant growth, development, and the enhancement of secondary metabolites and bioactive compounds, LEDs also find application as a non-chemical sanitation treatment. Although the sterilizing properties of UV radiation are well-established, recent studies have revealed that visible light can also exhibit bactericidal effects under specific conditions, highlighting its potential role in ensuring food safety [11]. A recent study demonstrated that plants exposed to high light intensity before harvest exhibited higher initial levels of carbohydrates and ascorbic acid that were maintained throughout the storage period. As a result, these plants exhibited improved visual quality and a longer shelf-life compared to other treatments [23]. In this context, the concepts of eustress, the beneficial and low-dose stress that enhances plant quality and productivity, and hormesis are gaining attention in agriculture as strategies to improve crop quality and sustainability under climate challenges, particularly through physical stressors like specific LED light wavelengths [24,25].

Lamb’s lettuce (*Valerianella locusta* L.) is a fresh leafy vegetable renowned for its abundance of phenolic compounds and remarkably high folate content [26]. Additionally, it is rich in many other pro-health nutrients (e.g., ascorbic acid and omega-3 fatty acids) and it is characterized by exceptional taste qualities which make it appealing to consumers. Lamb’s lettuce plants were previously successfully investigated in LED light experiments [27,28,29]; however, the effects at the post-harvest and phenolic profiling of this light growing condition was still to be investigated.

Thus, this work focused on the study of the R:B (70%:30%) LED light treatment impacts on lamb’s lettuce. Indeed, this R:B LED combination was previously demonstrated as optimal for phenolic compounds and chemical composition of lamb’s lettuce [27,30]. The growth of lamb’s lettuce in control and R:B LED conditions was investigated from the morphological and biochemical point of view. Moreover, the potential enrichment of bioactive compounds was evaluated in plants and the R:B LED light impact on the quality and stability of the fresh-cut product thereof.

## 2. Results

### 2.1. Plants Growth Evaluation

**Figure 1:**
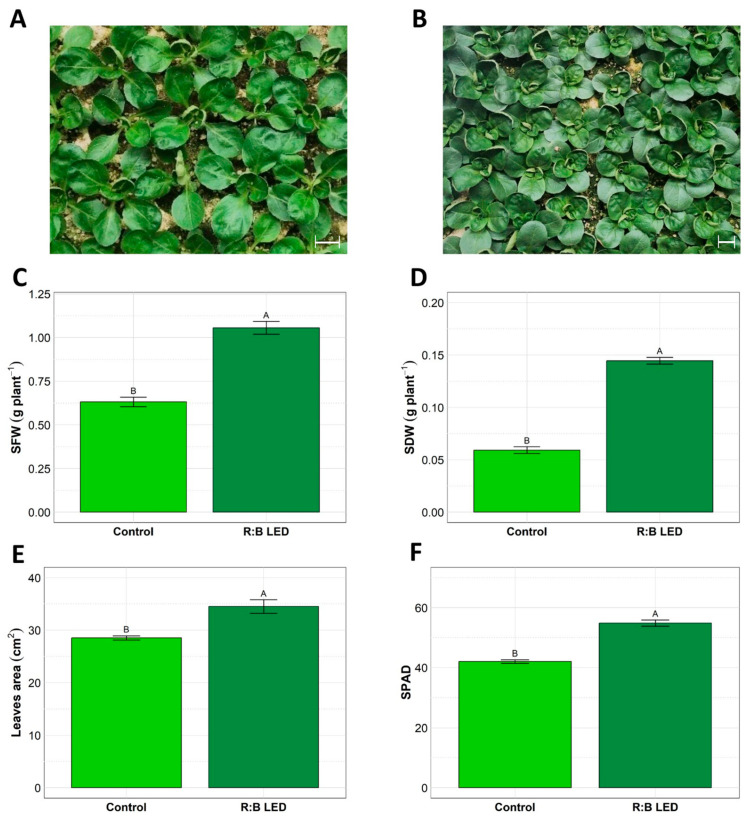
*Valerianella locusta* L. growth parameters. Representative pictures of *V. locusta* plants grown in control conditions at harvest (**A**); representative pictures of *V. locusta* plants grown in R:B LED light conditions at harvest (**B**). White bars in the pictures represent 1 cm length. Fresh biomass accumulation (**C**), dry biomass accumulation (**D**), leaf area development (**E**), and SPAD index (**F**) in *V. locusta* plants grown in either R:B LED light or control conditions at harvest. Data are reported as mean ± SE (n = 4 for SFW and SDW, n = 10 for SPAD index). The statistical significance has been tested through one-way ANOVA and Tukey post hoc test (*p* value < 0.05). Different letters indicate significantly different values.

In Figure 1 is depicted the visual comparison between *V. locusta* plants grown under control (white LED light) (Figure 1A) and R:B LED light (Figure 1B) conditions. Notably, the LED group exhibited a distinct darker green coloration and appeared larger overall compared to the control group. These visible differences were further confirmed by biometric parameters which were evaluated at harvest time (Figure 1).

The R:B LED light significantly impacted the *V. locusta* shoot fresh weight (SFW) and shoot dry weight (SDW) (Figure 1C,D). Indeed, treated plants showed 133% and 68% increase in SFW and SDW, respectively, suggesting the positive effect of R:B LED lights on the biomass accumulation. This was further confirmed by the leaf area (LA) evaluation (Figure 1E). Treated plants showed a statistically significant 21% increase in LA compared to the control ones. Furthermore, the mean SPAD index was significantly higher in LED-treated plants (54.86) as compared to control plants (42.06) (Figure 1F).

### 2.2. Organic Acids, Sugars, and Phenolic Compounds Concentrations

**Figure 2:**
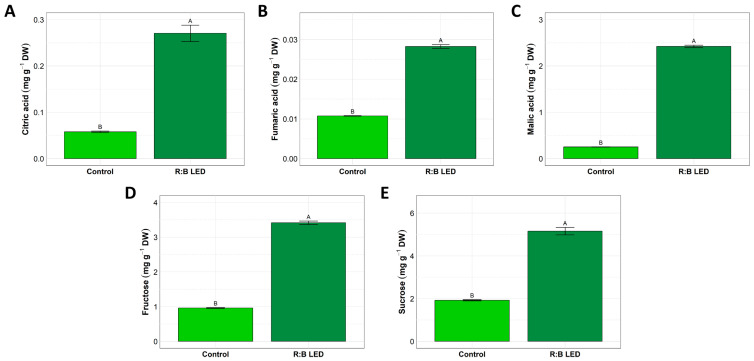
Organic acids and sugars quantification. Concentration of citric acid (**A**), fumaric acid (**B**), malic acid (**C**), fructose (**D**), and sucrose (**E**) in *V. locusta* plants grown in either R:B LED light or control conditions at harvest. Data are reported as mean ± SE (n = 6). The statistical significance has been tested through one-way ANOVA and Tukey post hoc test (*p* value < 0.05). Different letters indicate significantly different values.

*V. locusta* samples did not show any detectable peak ascribable to aconitic acid, oxalic acid, and succinic acid. On the other hand, the analysis of citric, fumaric, and malic acids indicated significant variations in the concentrations of these organic acids between the control and R:B LED-treated plants (Figure 2A–C).

In fact, R:B LED-treated plants exhibited a statistically significant increase in the concentration of citric acid (Figure 2A), fumaric acid (Figure 2B), and malic acid (Figure 2C) by about 350%, 200%, and 868%, respectively, as compared to control plants.

Similarly, *V. locusta* plants were analysed for the fructose, glucose, and sucrose content. Glucose was not detected in the samples analysed. However, treated plants exhibited a statistically significant increased concentration of fructose and sucrose compared to control plants. In detail, LED light-treated lamb’s lettuce showed an increment by about 255% and 169% of fructose and sucrose, respectively (Figure 2D,E).

As detected for the other metabolites, also phenolic compounds showed a differential accumulation depending on the light conditions. In fact, *V. locusta* plants subjected to R:B LED lights featured a 30.2% higher phenolic compound concentration with respect to control plants (Table 1).

### 2.3. Untargeted Phenolic Compounds Profiling

**Figure 3:**
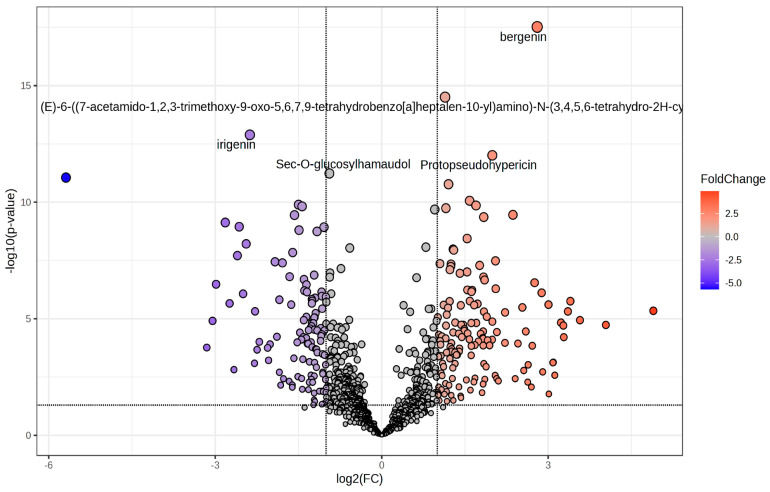
Volcano plot representing the significantly changed metabolites in *V. locusta* L. plants in R:B LED light conditions. The metabolites defined as significantly changed were those with log_2_(FC) > 1 and *p* value < 0.05, corrected by false discovery rate. The grey dots represent the metabolites with no significant differences; the red dots describe the increasing metabolites while the blue dots show the decreasing.

The effect of R:B LED light on the phenolic compounds composition of *V. locusta* plants was evaluate through UHPLC-HRMS untargeted phenolic compounds profiling. This approach allowed to putatively annotate 991 secondary compounds with 258 significantly modulated (*p* value < 0.05) in R:B LED-treated plants compared to the control with a |log_2_FC| ≥ 1 (Figure 3 and Supplementary Table S1).

**Figure 4:**
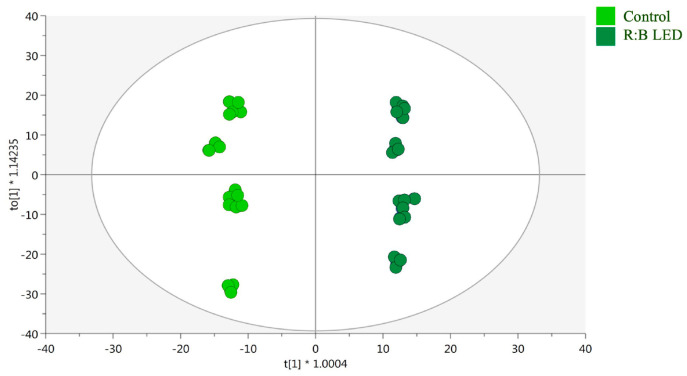
Supervised orthogonal projection to latent structures discriminant analysis (OPLS-DA). Score plot of the OPLS-DA carried out on untargeted metabolomics profiles of *V. locusta* plants growth in control and R:B LED conditions (R^2^Y = 0.99, Q^2^Y = 0.96). Each dot represents an individual technical replicate (n = 3) derived from each of the six biological replicates per treatment group.

Moreover, supervised OPLS-DA was performed to determine the markers of the differences noted in the control and R:B LED plants. Above all, as reported in the OPLS-DA score plot (Figure 4), the separation of R:B LED light-treated and control samples was observed on the latent vector t [2]. The model parameters were adequate, being goodness of fit (R^2^) = 0.99 and prediction ability (Q^2^) = 0.961 with no outliers (Hotelling’s T2). The CV-ANOVA (*p* value = 4.68 10^−17^) for regression and the permutation test (n = 100), excluded overfitting (Supplementary Table S1). Then, the variable importance in projection (VIP) method was used for the identification of the most discriminant compounds involved in the differences outlined by the OPLS-DA plot. Using a VIP score > 1.2, 246 compounds were selected, with kaempferol possessing the highest discrimination ability (VIP score = 1.8), followed by the isoflavan glyasperin D (VIP score = 1.78) and the hydroxybenzoic acid 2-hydroxybenzoic acid with a VIP score = 1.77.

**Figure 5:**
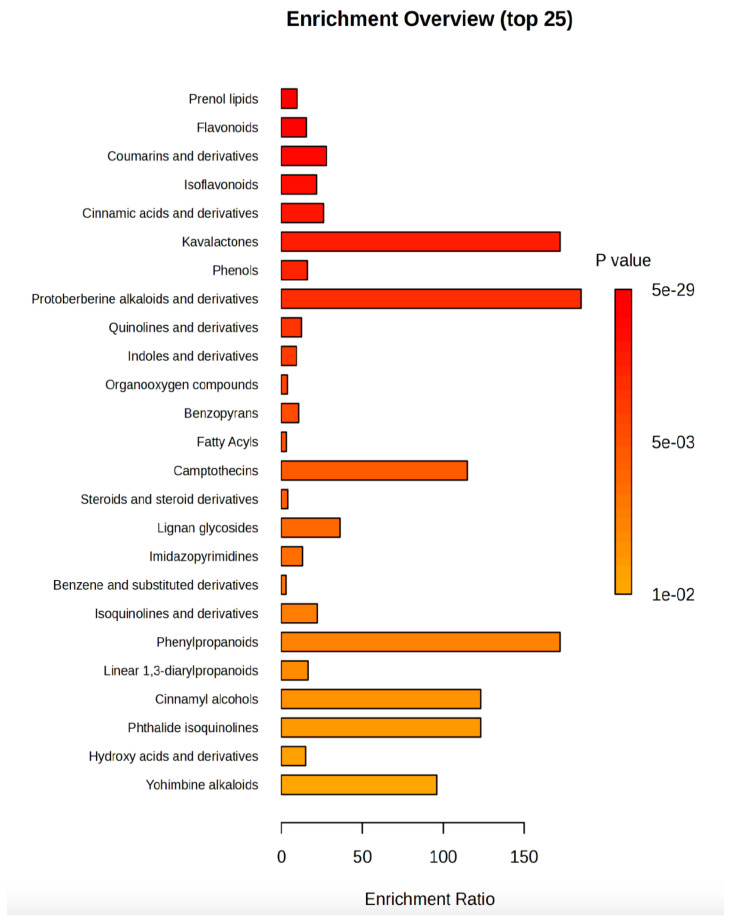
Metabolites enrichment analysis. The metabolites diagram displayed the 25 significantly (*p* value < 0.05) enriched classes of compounds in R:B LED light-treated *V. locusta* plants. The enrichment ratio is determined by the number of observed hits divided by the expected number of hits.

Finally, an overview of the most enriched classes of secondary metabolites (Figure 5) pointed out that kavalactones and protoberberine alkaloids and their derivatives were the classes with the lowest *p* value and the enrichment ratio higher than 50, followed by camptothecins, phenylpropanoids, cinnamyl alcohols, phthalide isoquinolines, and yohimbine alkaloids.

### 2.4. Antioxidant Properties

In this study, the antioxidant activities were evaluated using four different methods: 1,1-Diphenyl-2-picrylhydrazyl (DPPH), 2,2’-Azino-bis (3-ethylbenzothiazoline-6-sulfonic acid) (ABTS), Cupric Reducing Antioxidant Capacity (CUPRAC), and Ferric Reducing Antioxidant Power (FRAP) assays (Table 1). In general, independently from the assay considered, the antioxidant capacity of plants grown under R:B LED light resulted higher compared to control plants. In detail, DPPH scavenging activity resulted 12% higher in R:B LED-treated plants as compared to control plants, whereas slightly higher antioxidant ability, ranging between 42% and 45%, was assessed by ABTS, CUPRAC, and FRAP assays in *V. locusta* plants grown under R:B LED light. Also, the phosphomolybdenum assay (PBD), generally used to measure the total antioxidant capacity, resulted in a significantly higher activity (+18%) in lambs’ lettuce grown under R:B LED light with respect to control plants (Table 1).

Consistently, the determination of metal-chelating activity (MCA), applied to assess the ability of a sample to bind metal ions and prevent their catalytic activity, displayed higher values (+12%) in the case of R:B LED light-treated plants with respect to control ones (Table 1). The variability in the extent of responses observed among the assays can be attributed to the different chemical principles and sensitivities of each method. In fact, each antioxidant assay assesses distinct mechanisms of action and target specific classes of antioxidant compounds.

### 2.5. Enzyme Inhibitory Activity

The inhibitory potential of *V. locusta* extracts against cholinesterases (AChE and BChE), α-amylase, α-glucosidase, and tyrosinase enzymes was investigated (Table 2).

The biochemical assays did not show any significant difference between R:B LED light-treated plants and control ones when AChE, BChE, tyrosinase, and α-amylase inhibitory activity were considered. On the other hand, *V. locusta* plants exposed to R:B LED light featured a significant increase, by about 300%, in α-glucosidase inhibition with respect to control plants (Table 2).

### 2.6. Quality Evolution of Ready-to-Eat Lamb’s Lettuce During Refrigerated Storage

The lamb’s lettuce weight loss during the storage in refrigerated conditions was around 1–2% for both control and R:B LED-treated plants and was unchanged throughout the entire storage time. Similarly, firmness was comparable for both samples, accounting for ~0.72 kN during up to 28 days at 4 °C.

**Figure 6:**
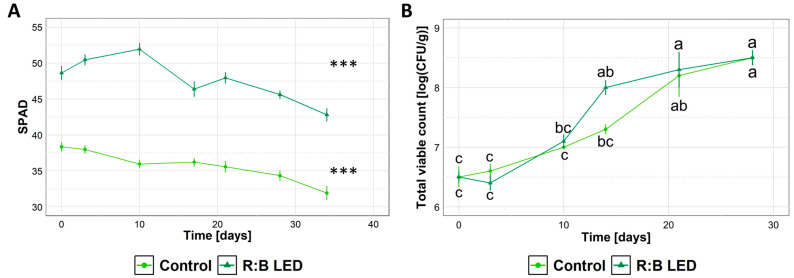
Characteristics of ready-to-eat *Valerianella locusta* L. upon cold storage. SPAD Index (**A**) and total viable count (TVC) (**B**) of ready-to-eat *V. locusta* grown in R:B LED light and control conditions at harvest. Data were collected from the harvest through 28 days for TVC and 34 days for SPAD of storage at 4 °C. SPAD index data are reported as mean ± SE (n = 10). TVC data are reported as mean ± SE (n = 3). The statistical significance has been tested through one-way ANOVA and Tukey post hoc test (*p* value < 0.05). Asterisks indicate significant difference upon storage within the same growing condition. Different letters indicate significantly different values.

Regardless of the cultivation system, the SPAD index progressively decreased upon storage (Figure 6A) indicating a loss of chlorophyll content of samples. Upon storage of lamb’s lettuce, no alterative events (i.e., rotting, browning) were visually detected. Similarly, no off flavours were detected upon informal sniffing, indicating that overall sensory attributes of lamb’s lettuce were preserved. Independently of the exposure or not of lamb’s lettuce to R:B LED light, microbiological analysis revealed that samples did not support the growth of coliforms, which were found to be below 5 CFU/g for the entire storage time. Just after preparation, the total viable count (TVC) (Figure 6B) was 6.5 log CFU/g for both samples, in agreement with Manzocco et al. (2021) [31] who reported similar results for the fresh-cut product obtained from *V. locusta*. Upon cold storage, a progressive increase in TVC was detected, reaching 8.5 log CFU/g after 28 days, independently of the cultivation system.

## 3. Discussion

### 3.1. Plant Morphology and Growth Parameters

The scrutiny extended to the biometric parameters of shoot fresh weight (SFW) and shoot dry weight (SDW) of *V. locusta* plants yielded insights into growth dynamics showing that R:B LED treatment had a significant impact on plants growth. Additionally, an intriguing trend emerged from the SPAD measurement data, showing that the mean SPAD index increased in R:B LED-treated plants as compared to control plants. Similarly, the R:B LED treatment induced a significant increase in the total leaf area per plant with respect to control plants. Overall, these findings emphasized that R:B LED light can significantly stimulate the growth of *V. locusta* plants also inducing a significant accumulation of pigments at the leaf level.

Plant biomass holds substantial importance as a critical growth parameter and it is conventionally regarded as a dependable marker for evaluating a plant’s resilience or vulnerability to stress factors [32]. The plant’s morphogenetic responses, like shoot branching or tillering, stem elongation, and flowering induction, are significantly influenced by the surrounding light conditions [33]. The spectral composition of light emerged as the dominant factor in influencing stem elongation and subsequently causing more significant variations in plant height, overshadowing the role of light intensity [33]. Sequential and coordinated interplay of various photosensors [34], phytochromes (A and B), which are susceptible to red and far-red light, as well as phototropins and cryptochromes, which detect blue light, collectively contribute to the inhibition of stem elongation [35]. Notably, the interaction of red and blue LED irradiation has proven to be a catalyst for shoot organogenesis enhancement in a variety of species, including potato [36] and lilium bulb formation [37]. In addition, the augmentation of shoot regeneration has been documented in different species through monochromatic red or blue LED irradiation [38,39]. Moreover, our findings also align with other shoot elongation results induced in plants like blueberries [40] and sugarcane [41].

### 3.2. Content of Organic Acids, Sugars, and Phenolic Compounds

Malic, citric, and fumaric acid levels were measured for this study. Their concentrations are intricately regulated by phosphoenolpyruvate carboxylase (PEPC), citrate synthase (CS), and malate dehydrogenase (MDH), through either direct or indirect mechanisms [42]. Interestingly, there are few studies that mention the impact of LED light on organic acid metabolism. Nevertheless, it is well known that the concentration of organic acids in plants fluctuates in response to diverse environmental and developmental factors. These factors encompass variations in light and temperature, diurnal and seasonal changes, the ripening of fruits, the aging process, the specific plant tissue and cell compartments, as well as a broad spectrum of environmental stressors [43]. In fact, the ultimate content of organic acids within a plant or specific tissue is dictated by the overall equilibrium between acid synthesis, degradation, utilization, and compartmentalization. Several factors including temperature, light exposure, fertilization, water availability, and various plant management techniques influence the source-to-sink ratio, consequently impacting the organic acid content [44]. However, another study that focused specifically on the metabolic changes of organic acids failed to provide conclusive data on the effect of light on the synthesis of citric acid, malic acid and fumaric acid in plants [45]. Therefore, the impact of light on organic acid metabolism is a subject of conflicting findings in the literature. The observed variations in organic acid concentrations of our study underline the potential influence of light quality on primary metabolic pathways in *V. locusta* plants, as large differences in citric, malic, and fumaric acid concentrations were observed between the two treatments considered. In fact, the specific light spectra we provided by LED supplementation appeared to trigger metabolic shifts, leading to altered organic acid profiles. These findings suggested a direct interaction between light perception and metabolic regulation, emphasizing the need for further research to understand the molecular mechanisms behind these observations.

The findings of this study further revealed the complex relationship between light quality, carbohydrate metabolism, and their implications for plant growth and development. Our results, which showed variations in sucrose and fructose levels under different light conditions, aligned with previous research that emphasized the pivotal role of light quality in shaping plant physiological processes. Interestingly, besides functioning as carbon (C) source and energy reservoir, sugars also play a crucial role in responding to oxidative stress and stabilization mechanisms, highlighting their sensitivity to environmental cues, including light quality [46]. Recent research conducted by Li et al. (2017) [47] shed light on the relationship between sucrose accumulation and the activation of specific antioxidant enzymes responsible for mitigating reactive oxygen species. In particular, Li et al. (2017) [47] also demonstrated that the exposure of tomato fruits to red light amplifies the activity of enzymes associated with sucrose metabolism, ultimately resulting in an elevated soluble sugar content. These observations were further confirmed in other plants such as Chinese bayberry [48].

In addition, numerous investigations have addressed the influence of light quality on plant C and nitrogen (N) metabolism. For instance, in cabbage, exposure to red LED light promotes the accumulation of carbon metabolites, including soluble sugars, sucrose, and starch [49]. Conversely, lettuce seedlings exposed to intermitting R:B LED lighting showed the highest levels of soluble sugars, starch, and sucrose, along with a higher C/N ratio, compared to those under R LED light alone [50]. On the other hand, it was observed that a 3 h supplementation of R:B LED light increased the expression of key Calvin cycle genes and sugar metabolism enzymes, enhancing CO_2_ fixation and photosynthetic capacity in cucumber plants [51]. The enzymes involved included invertases, sucrose synthases, and hexokinases. This treatment promoted the synthesis and accumulation of photosynthates in cucumber leaves [51].

The further biochemical profiling of *V. locusta* exposure to R:B LED light revealed a significant accumulation of phenolic compounds, suggesting that the light spectrum provided may have triggered a specific metabolic response. The synthesis of phenolic compounds follows the shikimate pathway in plants, where phenylalanine acts as a crucial intermediate. Interestingly, this pathway is regulated by several factors, among which light conditions and reactive oxygen species generated in response to excessive light exposure are well-characterized [52]. Indeed, earlier research has revealed that the combination of B and R LED lights, both individually [53] and concurrently [54], enhanced total phenolic content by increasing the photosynthetic activity and promoting the accumulation of malonyl-CoA, which is a precursor associated with phenolic compound synthesis.

### 3.3. Profiling of Phenolic Compounds

Untargeted phenolic compounds investigation demonstrated that *V. locusta* control samples and those subjected to R:B LED light had a completely different metabolic profile, as represented by the clear separation in the OPLS-DA score plot. One of the compounds that drove the differences between the two conditions was the secondary metabolite protopseudohypericin (VIP score = 1.70). This hydrated proto-form, together with protohypericin, are biosynthetic precursors that are subsequently converted into the cyclic compounds pseudohypericin and hypericin when exposed to light. These molecules have beneficial activity in neurodegenerative disorders due to their neuroprotective and antidepressant properties [55]. Thus, the quality and wavelength of light determine the synthesis of primary (proteins, carbohydrates, vitamins, etc.) and secondary metabolites which provided favourable properties to the cultivated agricultural and horticultural species [13]. Several studies reported the positive effect of R:B LED light on sugars content and antioxidant capacity, phenolic compounds, carotenoids, ascorbic acid, and chlorophyll in different leafy vegetable crops [56,57]. In fact, exposure to different wavelengths induces distinct physiological processes in plants leading to changes in pigments and their related biosynthetic enzymes [58]. These effects are mediated through specific photoreceptors such as phytochromes (red/far-red light) and cryptochromes or phototropins (blue light), which regulate downstream gene expression related to metabolic pathways [59].

Accordingly, in our results, compared to the control, the R:B LED light induced an over-accumulation of several flavonoid glycosides including kaempferol-4’-glucoside, naringin dihydrochalcone, baohuoside I, and engeletin. These compounds are well documented for their strong antioxidant, anti-inflammatory, and anticancer properties [60,61,62]. Modulation of isoflavones, gallic acid, coumaric acid, cinnamic acid, and triterpenoids was also observed. These compounds are likely key contributors to the enhanced antioxidant activity measured in R:B LED-treated samples. Notably, the first stage of the phenylpropanoids synthesis is light-dependent because the activity of phenylalanine ammonia-lyase (PAL) is regulated by the light; this enzyme catalyses the deamination of p-phenylalanine to trans-cinnamic acid. In addition, the expression and the activity of chalcone synthase, which condenses the CoA-ester of cinnamic acid with malonyl-CoA, is also regulated by light [58]. In particular, Długosz-Grochowska et al. (2017) [28] outlined that blue LED light has a major role in the regulation of polyphenol synthesis, with low concentrations of these compounds under lacks blue wavelengths radiation. Therefore, the result obtained from the volcano analysis outlined an increase in the alkaloid baptifoline (log_2_FC = 4.89) under R:B LED light. The biosynthesis of alkaloids is also influenced by light-mediated gene expression, as B and R light promote transcription of alkaloid pathway enzymes [63]. These compounds might possess a wide range of pharmacological properties including antitumor, antibacterial, and anti-inflammatory activities [64].

Likewise, our analysis suggested also an increase in terpenoids under combined R:B LED light. These results were in accordance with previous data [65], pointing out that a combination of R:B LED light induced an increase in terpenoid content in basil, achieving similar results in *Perovskia* [66]. A modulation of coumarins and derivatives was noted supporting earlier findings [67] and revealing that light stimulated the accumulation of coumarins in *Eclipta alba*. Together with these bioactive compounds, the enrichment analysis also suggested an increase in kavalactones including dihydrokawain, yangonin, and dihydromethysticin.

### 3.4. Antioxidant Properties and Enzyme Inhibitory Activity

The assessment of antioxidant activity through various assays provided valuable insights into the capacity of plant extracts to neutralize free radicals and oxidative stress. Our observations collectively suggested that the R:B LED light supplementation had the capability to enhance the antioxidant capacity of *V. locusta* plants, potentially leading to increased protection against oxidative stress. Indeed, phenolic compounds and flavonoids are known to possess strong antioxidant properties, and their presence in a sample can contribute to its overall antioxidant capacity as measured by these assays. The observed increase in phenolic content and antioxidant activity under R:B LED light aligns with and, in some cases, surpasses findings reported in similar studies. This work recorded a 30% increase in total phenolic content, which is comparable to or exceeds the 15–25% increases previously observed in lamb’s lettuce and basil under similar R and B LED light treatments [21,28]. In terms of antioxidant capacity, this study showed an enhancement of 12–45% across DPPH, ABTS, CUPRAC, and FRAP assays, which is consistent with prior findings on kale and spinach where antioxidant activity improvements ranged from 30–40% [20]. Moreover, Samuolienė et al. (2011) [68] found that the application of B and R LED light had the potential to enhance the antioxidant properties of sprouted lentil and wheat seeds, which was attributed to the higher levels of total phenols present in the sprouted seeds. However, besides phenolics, other compounds (e.g., carotenoids) can also contribute to the antioxidant activity [69].

In addition to antioxidant activity, enzyme inhibition also plays a central role in pharmaceutical and nutraceutical applications, as many diseases can be traced back to the activity of certain enzymes in the human body [70]. For this reason, the inhibitory potential of *V. locusta* extracts were evaluated against an array of key enzymes, namely cholinesterases (AChE and BChE), α-amylase, α-glucosidase, and tyrosinase. In particular, the combination of R:B LED light was shown to positively impact only the α-glucosidase inhibition activity. α-glucosidase inhibitors have gained significant pharmacological interest since they are widely used in the management of type 2 diabetes, particularly for mitigating postprandial hyperglycaemia by slowing down the breakdown of dietary carbohydrates [71]. The enhanced α-glucosidase inhibition observed under R:B LED light is likely due to the specific induction of secondary metabolites, such as flavonoids (e.g., kaempferol derivatives) and alkaloids (e.g., baptifoline), which are known inhibitors of this enzyme [72,73,74]. These compounds may act synergistically to modulate glycaemic response, providing a functional food benefit and suggesting that targeted light strategies could improve the antidiabetic potential of leafy vegetables. In contrast, enzymes like AChE and BChE are likely regulated by other metabolites that were not significantly modulated in our study, underscoring the specificity of light-induced metabolic pathways. This evidence encourages further investigation into the potential connections between light conditions and enzymatic modulation.

### 3.5. Post-Harvest Quality

Lamb’s lettuce is mainly consumed as a fresh-cut product, sealed in bags and stored at refrigeration temperature to guarantee adequate product shelf life. For this reason, *V. locusta* samples subjected to R:B LED light conditions were also processed into a fresh-cut product, which was evaluated for quality parameters upon a refrigeration period of 30 days. Our observations highlighted a loss of firmness and a decrease in SPAD index upon storage, which were indeed in accordance with previous data [31] showing the same downward trend when ready-to-eat lamb’s lettuce was stored at 4 °C for up to 30 days.

The detected microbial contamination level was expected since vegetables were not sterile. Cutting and washing operations, which are typically performed during fresh-cut processing, are well known to induce a moderate decrease in bacteria count solely [75]. The assessment of TVC during product storage was thus performed with the aim of assessing if the applied LED light pre-treatment could impair product stability during storage, modifying product shelf life. The observed increase in TVC upon storage can be attributed to the fact that minimally processed products are more prone to microbial multiplication than intact plants due to the presence of cutting surfaces, altered plant tissue respiration, and confinement in the packaging [75]. In this regard, some European countries suggest producers to adopt a specific microbiological limit, corresponding to 7 log CFU/g, to estimate the shelf life of minimally processed fruit and vegetables. According to this criterion, the 7 log CFU/g limit was exceeded at 14 days of storage for both control and R:B LED light-exposed plants. This period is in line with the average shelf life of fresh-cut vegetables, which has been reported to vary between 6 and 21 days depending on the product, packaging material, and storage temperature [76]. Indeed, these results demonstrated that hygienic quality should be considered as the main indicator of qualitative decay of fresh-cut *V. locusta* during refrigerated storage. In fact, microbial count grew beyond the acceptability limit much earlier than any changes in other quality parameters (i.e., SPAD index, weight loss, and firmness) were detectable, making it a key parameter for assessing the quality of *V. locusta.*

### 3.6. Conclusions

The study of red and blue (R:B, 70%:30%) LED light supplementation on *V. locusta* revealed significant effects on plant physiology and biochemistry. R:B light improved chlorophyll content, shoot and root biomass, and leaf area, indicating enhanced plant morphology. It also increased fructose and sucrose levels, suggesting a positive influence on carbohydrate metabolism and energy allocation.

Significant changes in citric and malic acids under R:B light highlighted their role in modulating primary metabolic pathways. Notably, the rise in phenolic compounds and associated antioxidant activity pointed out a strengthened in defense system and enhanced synthesis of bioactive metabolites, including flavonoids, alkaloids, and terpenoids.

Enzyme inhibition assays revealed a marked increase in α-glucosidase inhibition, suggesting potential antidiabetic properties. Lastly, post-harvest analysis showed that all light conditions supported acceptable shelf-life and microbiological quality in fresh-cut *V. locusta*.

Incorporating these findings into the broader context of plant science and controlled environment agriculture (e.g., vertical farming), this study underscored the significance of light quality in shaping plant biochemistry, paving the way for informed strategies in crop cultivation. Nevertheless, the significant variability of some results necessitates further analyses to understand the precise molecular mechanisms driving these observed phenomena. Overall, this research opened promising avenues for optimizing crop production to deliver nutrient-dense and high-quality products, with potential implications for both agriculture and human health.

## 4. Materials and Methods

### 4.1. Plant Growth Conditions

Lamb’s lettuce (*Valerianella locusta* L. cv Volhart 3) plants were cultivated in 104-well trays filled with a mixture of river sand (Laterlite Spa, Milano, Italy) and perlite (Orvital Spa, Milano, Italy) (1:1 *v*/*v*). Plant cultivation was carried out in fall season in the plant-growth chamber Microcosm (Piano Green Srl, Bolzano, Italy) equipped with LED lights. All plants, one per well, were cultivated for 36 days from seed sowing, with a photoperiod of 16/8 h (day/night) and a total photosynthetic photon flux density (PPFD) of 200 µmol m^−2^ s^−1^. Control plants were illuminated for the whole growth period with white (350–750 nm) LED light (Supplementary Figure S1A), whereas treated plants were illuminated for the first 14 days with white LED light, followed by 22 days of red (645–695 nm) LED light and blue (425–475 nm) LED light mixed in a ratio of 70%:30% red/blue (R:B) (Supplementary Figure S1B). The Microcosm maintained the average temperature at 20 °C, with a relative humidity (RH) of 56% and a ventilation rate of 10 air changes per hour (ACH). All plants were irrigated three times per week with 10 mL of a modified Hoagland’s solution with the following composition: 0.36 g L^−1^ Ca(NO_3_)_2_, 0.1 g L^−1^ KH_2_PO_4_, 0.13 g L^−1^ MgSO_4_, 0.8 g L^−1^ KNO_3_, 0.04 g L^−1^ NH_4_NO_3_, and 0.01 mg L^−1^ Mikron fertilizer (Cifo Srl, Bologna, Italy).

### 4.2. Plant Growth Evaluation

On the 36th growth day, four plants of each treatment were considered for leaf area (LA) evaluation. All the plant leaves were digitally scanned utilizing Easy Leaf Area software (version 2.0) [77], using as reference 1 cm^2^ square and default parameters.

The same plants were considered for shoot fresh weight (SFW) and subsequently dried at 65 °C until constant weight to determine the shoot dry weight (SDW).

Additionally, the SPAD index of fully expanded leaves of ten plants of each treatment was assessed using a SPAD-502 portable chlorophyll meter (Minolta, Osaka, Japan). The average of five SPAD measurements was calculated for each leaf.

### 4.3. Preparation of Fresh-Cut Lamb’s Lettuce

Lamb’s lettuce leaves were immediately processed after harvesting. In particular, leaves were washed with water at 8 °C for 3 min with a salad-water ratio of 1:18 (*w*/*w*) and then centrifuged in a manual kitchen centrifuge for 1 min. Aliquots of 20 g of lamb’s lettuce were packed under air in 30 × 20 cm bags of a commercial bioriented polypropylene (BOPP, 30 µm thickness) (Taghleef Industries SPA, S. Giorgio di Nogaro, Italy), sealed by a packaging machine (Easy Packer EP-400-C; AVC Italia, Turin, Italy), and stored at 4 °C for increasing times up to 28 days.

### 4.4. Organic Acids and Sugars Content

Organic acids (aconitic, citric, fumaric, malic, oxalic, and succinic acids) and sugars (fructose, glucose, and sucrose) were quantified according to Pii et al. (2018) [78] and Valentinuzzi et al. (2018) [79]. Briefly, freeze-dried leaves of six biological replicates of each treatment were extracted with methanol (HPLC grade, Merck, Darmstadt, Germany) using a 1:10 extraction ratio. Samples were then sonicated for 30 min in a thermostatic bath and centrifuged at 14,000× *g* for 30 min at 0 °C. Finally, the supernatant was collected and filtered through a 0.2 μm filter (Sarstedt AG, Nümbrecht, Germany). The compound separation was carried out through HPLC using a cation exchange Aminex 87-H column (300 × 7.8 mm, 9 μm, Bio-Rad), employing an isocratic elution with 10 mM H_2_SO_4_ as the carrier solution at a flow rate of 0.6 mL min^−1^. Detection of organic acids occurred at 210 nm utilizing a Waters 2998 photodiode array detector (Waters Spa, Milano, Italy), while sugars were detected via a refractive index detector (Waters Spa, Milano, Italy). Standard acids and sugars from Sigma Aldrich (St. Louis, MO, USA) were used to produce calibration curves for each molecule considered.

### 4.5. Total Phenolic Compounds Content

Four biological replicates of each treatment were used for the determination of total phenolic compounds leaf content. Total phenolic content evaluation was conducted as reported in Uysal et al. (2017) [80]. A sample solution (0.25 mL) was vigorously combined with a diluted Folin–Ciocâlteu reagent (1 mL, 1:9, *v*/*v*). After 3 min, a Na_2_CO_3_ solution (0.75 mL, 1%) was added to the mixture. The absorbance was measured at 760 nm following a 2 h incubation at room temperature. The total phenolic content was calculated in milligrams of gallic acid equivalents per gram of extract (mg GAE g^−1^).

### 4.6. Untargeted Phenolic Compounds Profiling by HRMS Metabolomics

The lamb’s lettuce metabolites were extracted by six biological replicates in a hydro-alcoholic solution (80% methanol, *v*/*v*) acidified with 0.1% formic acid with a homogenizer (Polytron PT 1200 E, Kinematica AG, Lucerne, Switzerland). The extracts were then centrifuged (6000 × g for 15 min at 4 °C) and filtered with 0.2 μm cellulose syringe filters into vials. The UHPLC-HRMS analysis was performed through a Q Exactive™ Focus Hybrid Quadrupole-Orbitrap Mass Spectrometer (Thermo Scientific, Waltham, MA, USA) coupled to a Vanquish ultra-high-performance liquid chromatography (UHPLC) pump, equipped with a heated electrospray ionization (HESI)-II probe (Thermo Scientific, USA). The mobile phases were water and acetonitrile (both LC-MS grade, from Sigma-Aldrich, Milan, Italy), gradient elution from 6 to 94% acetonitrile in 35 min, and as phase modifier 0.1% formic acid. The analytical column used for chromatographic separation was an ACQUITY UPLC BEH C18 (2.1 × 100 mm, 1.7 μm). The MS analysis was in full scan mode (in the m/z range 80–1200) with positive ionization (mass resolution: 70,000 at m/z 200), flow rate of 200 μL/min, and injection volume of 6 μL. Moreover, n = 3 quality control (QC) samples were injected randomly and analysed in a data-dependent (top n = 3) MS/MS mode, with the fragmentation of the most abundant ions under stepped normalized collisional energy (i.e., 10, 20, 40 eV).

Subsequently, the raw data were handled in the software MS-DIAL (version 4.90) [81], according to automatic peak finding, locally weighted scatterplot smoothing (LOWESS) normalization, and annotation via spectral matching. The comprehensive FooDB (https://www.foodb.ca) database was exploited for annotation; identification was achieved by accurate mass tolerance, isotopic pattern, and spectral matching. Accordingly, a level 2 confidence in annotation (typical of untargeted metabolomics) was achieved, according to the COSMOS standards in metabolomics [82]. A confidence level of 2 implies putative compounds annotation without confirmation by authentic standards allowing a broad metabolic coverage, but leading to potential uncertainties in compound identification which is a common limitation of untargeted metabolomics [83].

### 4.7. Antioxidants and Enzyme Inhibitory Activities

The antioxidant potential was assessed using a range of complementary assays, including DPPH and ABTS radical scavenging assays (which measure the ability of compounds to neutralize free radicals through hydrogen or electron donation), reducing power assays (CUPRAC and FRAP, which evaluate the electron-donating capacity of antioxidants), phosphomolybdenum assay (PBD) (used to determine total antioxidant capacity, capturing both hydrophilic and lipophilic antioxidants), and metal-chelating activity (MCA) (which assesses the ability of compounds to bind transition metals such as Fe^2+^, thereby preventing the generation of reactive oxygen species via Fenton-type reactions). Enzyme inhibitory activities were also evaluated, including cholinesterase (AChE and BChE) via Elmann’s method, tyrosinase using the dopachrome method, α-amylase employing the iodine/potassium iodide method, and α-glucosidase through the chromogenic PNPG method. These assays were conducted on four biological replicates per treatment, following methodologies previously outlined [80,84]. DPPH, ABTS, CUPRAC, and FRAP assays were expressed as mg Trolox equivalents (TE) g^−1^ extract. Total antioxidant activity carried out through PBD assay was expressed as mmol TE g^−1^ extract, whereas the MCA was reported as mg EDTA equivalents (EDTAE) g^−1^ extract.

AChE and BChE inhibitory activities were given as mg galanthamine equivalents (GALAE) g^−1^ extract, tyrosinase inhibitory activity was expressed as mg kojic acid equivalents (KAE) g^−1^ extract, and amylase and glucosidase inhibitory activities were presented as mmol acarbose equivalents (ACAE) g^−1^ extract.

### 4.8. Weight Loss and Firmness

Weight loss of fresh-cut lettuce was determined by weighing the content of the packages before and after the storage period, whereas SPAD values were recorded regularly during the storage period.

The firmness of the lamb’s lettuce was measured by an Instron 4301 universal tester (Instron Ltd., High Wycombe, UK) using a ten-blade Kramer shear cell. The instrumental settings and operations were accomplished using the software Automated Materials Testing System (version 5, Series IX; Instron Ltd.). Aliquots of 10 g of lamb’s lettuce were compressed to 50 mm. The test speed was 50 mm/min. Force–distance curves were recorded and firmness was taken as the maximum force required to compress salad. The determination was performed at the beginning and end of storage.

### 4.9. Microbiological Analyses

About 10 g of fresh-cut lamb’s lettuce were homogenised in a Stomacher (International PBI, Milan, Italy) for 1 min at normal speed with 90 mL of maximum recovery diluent (MRD; Oxoid, Basingstoke, UK). Serial dilutions (1:10) were made in MRD and analysed for microbial counts. Appropriate aliquots (0.1 mL or 1 mL) were spread on agar plates. Plate count agar (Oxoid) was used for the enumeration of aerobic mesophilic bacteria at 30 °C for 48 h. Pour plating in Coli ID (bioMerieux, Mercy l’Etoile, France) with a covering layer of the same medium incubated at 37 °C for 24 h was used for the enumeration of total and faecal coliforms. The determinations were conducted at 0, 3, 10, 14, 21, and 28 days.

### 4.10. Statistical Analysis

Statistical significance analysis for each data assay was conducted applying one-way ANOVA test followed by Tukey’s HSD test with *p* value < 0.05 using R software (version 4.0.3). The following R packages were used for data visualization and statistical analyses: ggplot2, agricolae, and ggpbur.

For metabolomics data, both unsupervised and supervised statistical analysis were performed in MetaboAnalyst 5.0 and SIMCA 13 (Umetrics, Malmo, Sweden) software, respectively. The raw data were centred by median, log_2_ transformed, Pareto scaled, and used to build the hierarchical cluster analysis (HCA, using Euclidean distance and Ward’s method as the linkage rule) and the orthogonal projections to latent structures discriminant analysis (OPLS-DA), considering the control samples and R:B LED light-treated lettuces. Following the OPLS-DA model validation parameters, goodness-of-fit (R^2^) and goodness-of-prediction (Q^2^) were registered, the model was inspected for outliers, cross-validated (CV-ANOVA), and the permutation test (n = 100) was executed to exclude overfitting. Then, the compounds mostly responsible for discrimination between the condition tested were investigated through the variable importance of projection (VIP) analysis, using a VIP score > 1.1.

## Figures and Tables

**Table 1:** Quantification of total phenolic compounds and antioxidant activity in extracts of *V. locusta* L. plants grown in either R:B LED light or control conditions at harvest. Data are reported as mean ± SE (n = 4). The statistical significance has been tested through one-way ANOVA and Tukey post hoc test (*p* value < 0.05). Different letters indicate significantly different values.

	Treatments	
	Control	R:B LED
Total Phenolic compounds (mg GAE g^−1^)	33.61 ± 0.91 b	43.77 ± 1.24 a
DPPH (mg TE g^−1^)	62.29 ± 1.54 b	69.71 ± 0.22 a
ABTS (mg TE g^−1^)	104.34 ± 5.10 b	148.81 ± 9.82 a
CUPRAC (mg TE g^−1^)	120.59 ± 8.26 b	170.76 ± 6.16 a
FRAP (mg TE g^−1^)	78.73 ± 2.69 b	114.04 ± 5.86 a
PBD (mmol TE g^−1^)	1.79 ± 0.07 b	2.11 ± 0.12 a
MCA (mg EDTAE g^−1^)	25.56 ± 0.85 b	28.72 ± 0.96 a

**Table 2:** Quantification of enzyme inhibition activities in extracts of *V. locusta* L. plants grown in either R:B LED light or control conditions at harvest. Data are reported as mean ± SE (n = 4). The statistical significance has been tested through one-way ANOVA and Tukey post hoc test (*p* value < 0.05). Different letters indicate significantly different values.

Treatment	AChE (mg GALAE g^−1^)	BChE (mg GALAE g^−1^)	Tyrosinase (mg KAE g^−1^)	α-amylase (mmol ACAE g^−1^)	α-glucosidase (mmol ACAE g^−1^)
Control	1.77 ± 0.19 a	0.44 ± 0.20 a	55.44 ± 4.36 a	0.29 ± 0.01 a	0.08 ± 0.05 b
R:B LED	1.99 ± 0.11 a	0.51 ± 0.12 a	53.04 ± 3.32 a	0.28 ± 0.01 a	0.32 ± 0.08 a

## Data Availability

The authors declare that the data supporting the findings of this study are available within the paper and its Supplementary Information Files. Should any raw data files be needed in another format they are available from the corresponding author upon reasonable request.

## Supplementary Materials

The following Supporting Information can be downloaded at: https://www.mdpi.com/article/10.3390/plants14121887/s1. Table S1: List of significantly modulated metabolites in extracts of *V. locusta* plants grown in R:B LED light as compared to control conditions. Figure S1: Emission spectra of white LED light (A) and of R:B LED light (B).

## Abbreviations

The following abbreviations are used in this manuscript:

ABTS	2,2’-Azino-bis (3-ethylbenzothiazoline-6-sulfonic acid)
ACAE	acarbose equivalents
ACH	air changes per hour
AChE	acetylcholinesterase
BChE	butyrylcholinesterase
BOPP	bioriented polypropylene
CUPRAC	Cupric Reducing Antioxidant Capacity
DPPH	1,1-Diphenyl-2-picrylhydrazyl
EDTAE	EDTA equivalents
FRAP	Ferric Reducing Antioxidant Power
GAE	gallic acid equivalents
GALAE	galanthamine equivalents
HESI	heated electrospray ionization
HPLC	High-Performance Liquid Chromatography
KAE	kojic acid equivalents
LA	leaf area
LED	Light-Emitting Diode
MCA	metal-chelating activity
MNM	micronutrient malnutrition
MRD	maximum recovery diluent
PBD	phosphomolybdenum activity
PNPG	p-nitrophenyl-β-glucopyranoside
PPFD	photosynthetic photon flux density
R:B	red/blue
RH	relative humidity
SDW	shoot dry weight
SFW	shoot fresh weight
TE	Trolox equivalents
UHPLC-HRMS	ultra-high-performance liquid chromatography–high-resolution mass spectrometry
UV	ultraviolet
